# Evaluation of novel herbal antifungal efficacy against *Candida albicans* isolated from oral leukoplakia

**DOI:** 10.6026/973206300214901

**Published:** 2025-12-15

**Authors:** Neetu Pandey, Sweta Singh, Gopal Srivastava, Chirag Patel, Rangoli Srivastava, Sameer Gupta

**Affiliations:** 1 Department of Oral Medicine and Radiology, Mansarovar Dental College Madhya Pradesh, Bhopal, Madhya Pradesh, India; 2 Department of Oral & Maxillofacial Pathology and Oral Microbiology, Himachal Institute of Dental Sciences, Rampur Ghat Road Ponta Sahib, Himachal Pradesh, India; 3 Department of Oral Medicine and Radiology, Government Dental College, Thiruvananthapuram Medical College Campus, Kerala, India; 4 Department of Pedodontics and Preventive Dentistry, Faculty of Dental Science, Dharmsinh Desai University, Nadiad, Gujarat, India; 5 Department of Public Health Dentistry, Faculty of dental sciences, SGT University, Gurgaon, Haryana, India; 6 Department of Oral & Maxillofacial Surgery, Inderprastha Dental College and Hospital, Sahibabad, Ghaziabad, Uttar Pradesh, India

**Keywords:** *Candida albicans*, oral leukoplakia, herbal extracts, antifungal activity, *Allium sativum*, *Curcuma longa*, *Azadirachta indica*, biofilm inhibition

## Abstract

*Candida albicans* infection and growing antifungal resistance are often linked with the emergence of an oral leukoplakia, a potentially malignant lesion that necessitates the implementation of alternative treatment methods. Therefore, it is of interest to compare antifungal effectiveness of *Curcuma longa*, *Azadirachta indica* and *Allium sativum* extracts on oral leukoplakia lesions *C. albicans*. Agar diffusion, MIC, biofilm inhibition and time-kill assays were done with different concentrations of ethanolic extracts as control was nystatin. The extracts that had the highest activity in the form of largest inhibition zones, least MIC and greatest biofilm activity were *A. sativum*. Thus, its herbal extracts particularly have a good potential when used as adjunctive antifungal agents in management of oral leukoplakia.

## Background:

The most common potentially malignant disease of the oral cavity is oral leukoplakia, which is a largely white, questionable-risk oral plaque, which cannot be classified as any other identifiable lesion, with prevalence reported varying between different groups of people between 0.5 and 3.4% [1]. Malignant transformation rate ranges between 0.13 and 17.5 percent with respect to clinical and histopathology characteristics which highlight the clinical importance of such lesions [2]. *Candida albicans*, which is a dimorphic opportunistic fungal pathogen, has been suggested to play a role in the pathogenesis and further progression of oral leukoplakia in several ways such as chronic inflammation, generation of carcinogenic nitrosamines, and promoting the penetration of tobacco carcinogens into the epithelial tissues [3]. Research has revealed colonization by *C. albicans* in 40-80 percent of oral lesions of leukoplakia and higher malignant transformation rates have been found in candidal leukoplakia than in non-candidal leukoplakia [4]. More so, fungal hyphae exhibit a higher tissue invasion ability, which may contribute to dysplastic alteration and malignant development [5]. The main current treatment of candidal infections in oral leukoplakia is based on the normal antifungal agents such as polyenes (nystatin, amphotericin B), azoles (fluconazole, ketoconazole), and echinocandins [6]. Nevertheless, the escalating antifungal resistance, side effects related to the long-term systemic treatment, drug interactions, and the high cost of the treatment are becoming major clinical challenges [7]. The development of azole-resistant Candida strains (especially in immunocompromised patients and in those receiving long-term prophylaxis of antifungal agents) has led to the need to develop alternative treatment approaches [8]. Plant-based herbal medicines have been in use throughout the millennia in various traditional medical systems such as Ayurveda, Traditional Chinese Medicine, and indigenous healing systems [9]. Modern scientific research has confirmed the antimicrobial activity of many botanical extracts and a number of them are broad-spectrum in action against bacterial, fungal, and viral microbes [10]. The benefits of herbal therapeutics are that they have multi-target mechanism of action which helps reduce resistance development, good safety profiles, availability and low cost [11].

The plant *Curcuma longa* (turmeric) is a rhizomatous herbaceous perennial belonging to the family of Zingiberaceae and it is a source of curcumin, a primary bioactive polyphenolic compound. Past studies have established the antifungal action of curcumin by disrupting the membrane, inhibiting ergosterol biosynthesis, and generation of reactive oxygen species [12]. *Azadirachta indica* (neem), a Meliaceae family of plant, includes azadirachtin and nimbidin, which have been shown to have antimicrobial and immunomodulatory effects [13]. *Allium sativum* (garlic), a plant that belongs to Amaryllidaceae, synthesizes allicin during tissue perturbation by alliin metabolism to allicin, and confirmed mechanisms of antifungal action are interference with cell wall synthesis and dysfunction of mitochondria [14]. Although antimicrobial properties of these herbal extracts have been reported, the overall assessment of the antifungal activity of the extracts against *C. albicans* isolates though not specifically against oral lesion of leukoplakia has not been carried out. Majority of past research has analyzed laboratory reference strains/isolates of other body locations and this may not reflect the specific resistance patterns and virulence traits of Candida in relation to mouth on potentially malignant disorders [15]. Besides, comparative evaluation of various herbal extracts with standardized methodological approaches and assessment of the biofilm-inhibitory effect - a crucial virulence factor in chronic candidal infections needs to be put under a systematic study. Therefore, it is of interest to assess in a comprehensive manner the antifungal activity of ethanolic extracts of *Curcuma longa*, *Azadirachta indica* and *Allium sativum* to *Candida albicans* isolates found in oral leukoplakia lesions. Part of the objectives was: (1) to establish zones of inhibition and MIC; (2) to determine the comparison between antifungal potency and traditional nystatin treatment; (3) to establish biofilm formation inhibitory capacity; (4) to determine time-kill kinetics; and (5) to identify significant phytochemical components of antifungal activity. Our hypothesis was that such herbal extracts would exhibit a high level of antifungal activity that can be used in clinical therapeutic or preventive use.

## Materials and Methods:

## Design and ethical approval of the study:

This laboratory case study was done in the period of January 2024-September 2024. Clinical collection of Specimens and Isolation of Fungi.

## Selection of patients:

The patients who were selected belonged to the Oral Medicine Department where they were recruited based on clinical examination and histopathological diagnosis of oral leukoplakia.

## Inclusion criteria:

Oral leukoplakia, clinically diagnosed, with the confirmation of a biopsy; age between 18-75 years; lack of antifungal treatment within the last 30 days. attained the age of eighteen years; no systemic immunosuppressive disease or condition (HIV, diabetes mellitus, organ transplantation); not taking antibiotics or antifungal drugs within 30 days; not pregnant or breastfeeding.

## Sample collection:

Surfaces of lesions of leukoplakia were sampled with sterile cotton swabs in accordance with the standard procedures. Immediately, swabs were placed in sterile tubes with Sabouraud dextrose broth (SDB) and ferried to the microbiology laboratory in a period that did not exceed 2 hours. Fungal isolation and identification: Samples were inoculated in the Sabouraud dextrose agar (SDA, HiMedia, India) added with chloramphenicol (50 mg/L) and labels incubated at 37°C over a period of 48 hours. Purification of suspected Candida colonies was done by subculture. Identification methods used: (1) Gram staining which indicated the presence of gram positive budding cells; (2) germ tube test which was positive after 2-3 hours incubating at 37C in human serum; (3) germ tube could be grown in CHROMagar Candida which showed green colonies (indicated *C. albicans*); (4) carbohydrate assimilation using API 20C AUX system (bioMerieux, France). Confirmed *C. albicans* isolates were stored in SDB containing 20% glycerol at -80°C. Herbal Extracts Preparation of Plant material sourcing: Certified dried rhizomes of *Curcuma longa*, Azadarachta indica leaves, and *Allium sativum* bulbs were purchased on the basis of certified suppliers. The Department of Pharmacognosy carried out botanical verification and stored voucher specimens (CL-2023-01, AI-2023-02, AS-2023-03).

## Preparation of extracts:

Plant materials were properly washed, dried under shade and fine powdered using mechanical grinder. Ethanolic extraction used Soxhlet equipment: 100g powdered material was extracted in 500ml of 95% ethanol and took 72 hours at 60°C. Whatman No. 1 filter paper was used to extract, rotary evaporator was used to concentrate under low pressure at 45°C and vacuum desiccator was used to dry extracts. End dried extracts were frozen at 4°C and in amber bottles.

## Percentage yield was obtained:

*C. longa* (12.4%), *A. indica* (8.7%), *A. sativum* (15.2%).

## Preparation of stock solution:

Stock solutions were prepared by serial dilution in sterile distilled water, 200 mg/ml of dried extracts in dimethyl sulfoxide (DMSO, 10% v/v). The total concentration of DMSO in assays was not more than 1% that was not antifungal in control experiments.

## Antifungal susceptibility testing:

## Agar well diffusion method:

Wells (8mm diameter) were prepared on Mueller-Hinton agar plates to which 2% glucose and 0.5 ug/ml methylene blue had been added. *C. albicans* suspensions were uniformly swabbed in wells with 1-5x10 6 CFU/ml. Test extracts of various concentrations were added as 100ul of the wells (nystatin 100, 000 IU/ml positive control, and sterile water negative control). The plates were incubated at 37°C between 24 and 48 hours. The digital calipers were used to measure zones of inhibition (ZOI) in millimeters. Triple repetitions were done on the experiments. Determination of Minimal Inhibitory Concentration (MIC): Broth microdilution experiment was conducted according to Clinical and Laboratory Standards Institute (CLSI) guidelines M27-A3. In short, herbal extracts (0.78-200 mg/ml) or nystatin (0.125-64 µg/ml) were microplated in 96 wells at 100mu l RPMI-1640 medium in the serial two-fold dilution. Fungal suspension 100 µl solution (0.5- 2.5 x 10 3 CFU/ ml final concentration) was inoculated into the wells. There were growth control (inoculum only), and sterility control (medium only). The plates were incubated at 35°C with 48 hours. The lowest level observed spectrophotometrically at 600nm was called MIC and was the lowest level at which there was 80 percent growth inhibition relative to control. Minimum Fungicidal Concentration (MFC): Aliquots (20µl) of wells that did not show any visible growth in MIC assay were subcultured on SDA plates and incubated at 37°C in 48 hours. MFC was taken as the lowest amount of concentration with 99.9 percent reduction in CFU relative to starting inoculum.

## Biofilm inhibition assay:

The crystal violet staining technique was used to determine biofilm formation. The yeast nitrogen base medium containing 100mM glucose was inoculated with *C. albicans* suspensions (1x 10 6 CFU/ml) and dispensed (200ul/well) into 96 well polystyrene plates. At the concentrations of MIC, 1/2MIC, and 1/4MIC, herbal extracts were added. Planktonic cells were scraped off after 48-hour incubation at 37°C, three times of PBS was used to wash off the wells, and methanol was used to fix biofilms. It was incubated with the crystal violet (0.1%, 200 1) and the surplus solution was washed off after 15 minutes followed by the solubilization of the bound dye in 33% acetic acid. The absorbance was taken at a wavelength of 570nm. The percentage of biofilm inhibition was determined: [(ODcontrol-ODtreatment)/ODcontrol) x 100].

## Time-kill kinetics:

Herbal extracts were subjected to fungal suspensions at concentration of 1x10 6 CFU/ml, 2xMIC, and 4xMIC. Aliquots (100uml) were harvested at 0, 2, 4, 8, 12, and 24 hours, serial diluted, placed on SDA and left to incubate to count the colonies. Plots were made of the results as log 10 CFU/ ml vs time. A fungicidal activity was considered that of 3 log 10 or higher reductions of CFU/ml.

## Phytochemical analysis:

Standard techniques were used in qualitative phytochemical screening to identify alkaloids, flavonoids, tannins, saponins, terpenoids, and phenolic. Waters Alliance system combined with C18 column, proper mobile phase, and UV detection at the designated wavelengths were used to quantify major bioactive compounds by the use of high-performance liquid chromatography (HPLC).

## Statistical analysis:

The SPSS version 26.0 (IBM Corporation) was used to analyze data. Descriptive statistics involved the mean standard deviation of continuous data and frequencies of categorical data. Shapiro-Wilk test was used to analyze normal distribution. One-way analysis of variance (ANOVA) was used to measure antifungal activity of extracts and concentrations, then, the honestly significant difference (HSD) post-hoc test was used to compare the results in pairs. Pearson correlation was used to determine the relationships between the extract concentrations and ZOI. The statistical significance was determined as p<0.05. Each of the experiments was conducted in triplicate with at least 60 isolates being examined.

## Results:

Sixty *Candida albicans* isolates were successfully obtained from 78 patients diagnosed with oral leukoplakia, yielding an isolation rate of 76.9%. All isolates were confirmed as *C. albicans* based on characteristic morphology, positive germ tube formation, green colony appearance on CHROMagar and API 20C AUX identification with 99% confidence. The study population had a mean age of 52.4 ± 11.8 years, with a male predominance (64%). The most common lesion site was the buccal mucosa (58%), followed by the lateral border of the tongue (23%), floor of the mouth (12%), and palate (7%). All three herbal extracts demonstrated a statistically significant concentration-dependent increase in antifungal activity against *C. albicans* isolates (one-way ANOVA, p < 0.001). Among the tested extracts, *Allium sativum* consistently produced the largest zones of inhibition at all concentrations. At the highest concentration tested (200 mg/ml), *A. sativum* exhibited a mean zone of inhibition of 24.3 ± 2.8 mm, which was not statistically different from that of nystatin (26.8 ± 1.9 mm, p = 0.087). *Curcuma longa* and *Azadirachta indica* showed lower but significant antifungal activity, with mean zones of inhibition of 21.7 ± 2.4 mm and 19.2 ± 2.1 mm, respectively, at 200 mg/ml.

Minimum inhibitory concentration analysis revealed that *A. sativum* had the lowest mean MIC value (12.5 ± 4.2 mg/ml), indicating superior antifungal potency compared to *C. longa* (18.7 ± 5.6 mg/ml) and *A. indica* (25.3 ± 6.8 mg/ml). The MIC of *A. sativum* was significantly lower than that of *C. longa* (p = 0.008) and *A. indica* (p < 0.001). Corresponding minimum fungicidal concentration values followed a similar trend, with *A. sativum* exhibiting the lowest mean MFC (25.0 ± 8.4 mg/ml). The MFC/MIC ratios for all herbal extracts were ≤4, confirming fungicidal activity. Distribution analysis of MIC values indicated that 88.3% of isolates exhibited *A. sativum* MIC values ≤25 mg/ml, compared with 76.7% for *C. longa* and 58.3% for *A. indica*. These findings further support the superior antifungal efficacy of *A. sativum* against *C. albicans* isolates. Biofilm inhibition assays demonstrated that all herbal extracts significantly reduced biofilm formation in a concentration-dependent manner. At MIC concentration, *A. sativum* achieved the highest mean biofilm inhibition (78.4 ± 6.3%), which was significantly greater than that observed with *C. longa* (64.2 ± 7.1%, p = 0.002) and *A. indica* (56.8 ± 6.9%, p < 0.001). The biofilm inhibitory effect of *A. sativum* was comparable to that of nystatin at MIC concentration (82.6 ± 5.4%, p = 0.241). Time-kill kinetic studies revealed concentration-dependent fungicidal activity for all herbal extracts. At twice the MIC, fungicidal effects were observed within 12 to 24 hours, whereas at four times the MIC, fungicidal activity was achieved more rapidly, within 6 to 16 hours, depending on the extract. *A. sativum* at four times the MIC demonstrated the most rapid fungicidal action, with a mean time to fungicidal effect of 6.8 ± 1.7 hours. Qualitative phytochemical screening confirmed the presence of flavonoids, alkaloids, tannins, saponins, and terpenoids in all herbal extracts. High-performance liquid chromatography analysis identified curcumin as the major bioactive compound in *C. longa* (78.4 mg/g of dry extract), azadirachtin in *A. indica* (34.6 mg/g), and allicin in *A. sativum* (156.8 mg/g). A strong positive correlation was observed between the concentration of these major bioactive constituents and antifungal potency, with correlation coefficients ranging from 0.87 to 0.94 (p < 0.001) (Table 1, 2 and 3 - see PDF).

## Discussion:

This thorough study shows that ethanolic extracts of *Curcuma longa*, *Azadirachta indica* and *Allium sativum* were significantly antifungal towards *Candida albicans* isolates derived as oral lesions of leukoplaka and that *A. sativum* has the strongest efficacy which is nearly similar to conventional therapy using nystatin. These results justify the possible clinical use of herbal extracts as alternative or adjunctive antifungal agents in the treatment of candidal infections related to the potentially malignant oral disorder [1]. The excellent antimicrobial effect of *A. sativum* (mean MIC 12.5 mg/ml, mean ZOI 24.3 mm at 200 mg/ml) is supported by the previous studies which have shown a strong antimicrobial activity of garlic extracts against different fungal pathogens [2]. Its action mainly relies on allicin, a sulfur-based compound, which is the resultant product of enzyme transformation of alliin through the action of alliinase on tissue damage. Allicin destabilizes the fungal cell membrane by interfering with thiol groups of key enzymes, inhibits ergosterol production and induces oxidative stress by the formation of reactive oxygen species [3]. The mode of high allicin concentration that was measured in our extract (156.8 mg/g) probably is the cause of the potency observed [4]. *Curcuma longa* had an important antifungal activity (average MIC 18.7 mcg/ml), which is consistent with previously reported antimicrobial action of curcumin and curcuminoids. Antifungal effects of curcumin involve disruption of membrane integrity, interference with cell wall synthesis, mitochondrial dysfunction and regulation of virulence factors such as biofilm formation, morphological transition between yeast and hyphae forms [6]. It is also of clinical significance that *C. longa* extract could inhibit 64.2% of biofilm formation at an MIC concentration, which is a significant virulence factor of chronic candidal infection and a significant contributor to antifungal resistance [7]. *Azadirachta indica* was found to have moderate antifungal activity (mean MIC 25.3 mg/ml) which even though has other extracts tested, was found to have higher therapeutic value. The bioactive compounds of neem, especially azadirachtin, nimbidin and gedunin, have antifungal properties, which work via various mechanisms such as the membrane permeabilization, enzyme inhibition and immunomodulatory [8 ]. Multi-target activity of the entire phytochemical profile of neem, comprising of more than 140 bioactive compounds, is likely to have synergistic effects on antimicrobial effects and minimize resistance formation [9]. The good biofilm inhibitory activity of *A. sativum* (78.4 percent at MIC) is a decisive benefit of clinical translation. The oral lesions of leukoplakia caused by *C. albicans* have up to 1000 times the resistance of conventional antifungals to *C. albicans* biofilms in planktonic cell formations, which results in a failure of treatment and persistence of disease [10]. An extract of herbs that can interfere with biofilm organization and maturation formation provide hope of dealing with these recalcitrant infections [11]. Time-kill kinetic analysis showed the fungicidal effect of all the extracts tested, with 4xMIC of *A. sativum* resulting in 3+ log 10CFU reduction in 6.8 hours. This quick fungicidal effect is clinically beneficial over fungistatic agents which only suppress growth because fungicidal action is able to decrease the burden of infection more effectively, as well as resistance development can be reduced [12]. The isolating rate of *C. albicans* in the oral lesions (76.9) is higher than those of other epidemiological studies that indicated 40-80% colonization rate [13]. Such correlation highlights the value of antifungal approaches in the overall management of leukoplakia since candidal elimination could lessen inflammation, lower the risk of malignant transformation, and enhance the rate of lesion healing [14]. These findings have clinical implications such as the possibility of the designing of herbal extract-derived topical preparations (mouthwashes, gels, lozenges) which have local antifungal effect in oral leukoplakia. The multi-targeted action of herbal compounds can bypass resistance of the conventional antifungals. Moreover, another possible benefit of anti-inflammatory and antioxidant effects of these extracts exists in the context of the treatment of potentially malignant disorders [15]. Lewis has significant drawbacks that should be mentioned. To begin with, this in vitro research cannot possibly generalize to clinical effectiveness because of the oral cavity circumstances such as fluctuation of pH levels and protein binding, enzyme degradation, and tissue infiltration, affect the drug activity. Second, the composition of herbal extracts is difficult to be standardized, as there is a difference in the genetics of plants, conditions of their growth and the method of their processing. Third, the research compared ethanolic extracts, it is not known if other extraction solvents or methods could produce different phytochemical profiles and activities. Fourth, they should be systematically evaluated with regard to long-term stability, toxicity, and allergic reactions before being used in clinical practice. Fifth, the paper concentrated on *C. albicans* only; activity against *non-albicans Candida spp* and mixed fungi infections are not studied. The limitations of this research should be resolved in future studies by employing randomised controlled clinical trials to assess clinical effectiveness and safety, and bioavailability and tissue penetration of pharmacokinetic studies, standardisation of extract composition, toxicology including mutagenicity and cytotoxicity tests, and possible synergistic effects of various herbal extracts or with a standard antifungal agent.

## Conclusion:

Ethanolic extracts of *Curcuma longa*, *Azadirachta indica* and *Allium sativum* demonstrated significant, concentration-dependent antifungal activity against *Candida albicans* isolates obtained from oral leukoplakia lesions. Among these, *Allium sativum* exhibited the greatest efficacy, showing fungicidal action, strong biofilm inhibition, and antifungal performance comparable to nystatin. These findings highlight the potential of selected herbal extracts as promising adjunctive antifungal agents, warranting further clinical and translational research for management of candidal involvement in oral leukoplakia.
